# Masterbatch-enabled acceleration of polyolefin biodegradation under open air terrestrial environmental conditions

**DOI:** 10.1038/s41529-026-00817-5

**Published:** 2026-08-11

**Authors:** Ceren Kütahya, Catalina Cruañas Pániker, Faith Ly, Arjun Jerath, Emma Little, Rocio Gali, Muhammad Zuhayr Bin Dzul Haimi, Abdul Hafiz Bin Abd Malek, Karimah Binti Muhamad, Suffeiya Binti Supian, Taylor B. Young, Sally Lawrence, Thomas Bell, Tan Yong Nee, José I. Jiménez, Florence Huynh

**Affiliations:** 1 Polymateria Limited, i-Hub, Imperial College White City Campus, London, UK; 2 Department of Life Sciences, Imperial College London, London, UK; 3 Standard and Industrial Research Institute of Malaysia (SIRIM), Shah Alam, Selangor Malaysia; 4 Department of Life Sciences, Georgina Mace Centre for the Living Planet, Imperial College, London, UK; 5 Imperial Centre for Engineering Biology, Imperial College London, South Kensington Campus, London, UK

**Keywords:** Biotechnology, Environmental sciences, Microbiology

## Abstract

Polyolefins, commonly used in packaging and single-use products, are notoriously persistent in the environment, contributing significantly to environmental pollution. In scientific literature to date, polyolefins have not been reported to fully biodegrade. This study examines the biodegradation potential of polyolefin materials, specifically polyethylene (PE) and polypropylene (PP), enhanced through the incorporation of Biotransformation Masterbatch technology. The inclusion of the Biotransformation Masterbatch accelerated and enabled the full biodegradation of PE and PP, as demonstrated by laboratory weathering, and biodegradation studies in soil at mesophilic temperatures. Ecotoxicity tests revealed no adverse effects on test organisms in both soil and water environments, while metagenomics analysis demonstrated that biodegradation of these polyolefins did not significantly change the soil microbiota composition, which showed higher metabolic activity compared to virgin plastic controls. These findings demonstrate that Biotransformation technology provides an effective solution for delivering polyolefin-based materials with reduced environmental impact. It offers a sustainable alternative to conventional plastics, preserving the performance characteristics of traditional polyolefins while addressing the problem with fugitive plastic waste in the environment.

**Figure:**
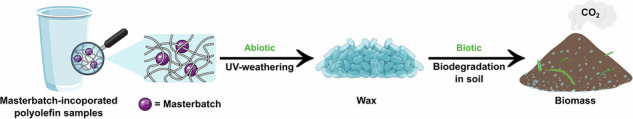

## Introduction

Polyolefin materials, such as polyethylene (PE) and polypropylene (PP), are the most widely produced and consumed synthetic polymers worldwide, with extensive applications in packaging, appliances, and single-use products^1–4^. Annual global plastics production now exceeds 400 million metric tons, approximately half of which consists of polyolefins^5,6^. Due to their chemical and biological inertness, these materials have high stability and resistance to (bio)-degradation. While these properties were originally regarded as advantageous, they have led to the persistent accumulation of plastic materials in the environment with 50% of globally produced plastic packaging escaping into nature^7–9^. Moreover, it is estimated that over 80% of plastic found in the oceans originates from land-based sources^10^. This underscores the urgent need for sustainable solutions addressing mismanaged end-of-life of plastic waste to mitigate its environmental impact.

As polyolefins continue to dominate commercial applications, reducing their environmental impact has become a major focus of scientific research. Polyolefins such as PE and PP are highly resistant to biodegradation due to several factors: their inert chemical structure, hydrophobic nature, typical high molar masses, and low bioavailability, where polyolefins typically form crystalline structures, which are less accessible to microbial degradation^11–14^. These inherent properties pose significant challenges to efforts aimed at reducing their environmental impact and thus enhance the need for the development of innovative solutions.

Efforts to modulate the biodegradability of polyolefins have led to extensive studies, but no strategy has been reported in the scientific literature to date yielding full biodegradation of these plastics in the open-air terrestrial environment. Recent research efforts have explored various strategies to overcome these obstacles, such as introducing functional monomers into the polymer structure or using microbial or enzymatic treatments to enhance degradation^13,15–17^. However, these methods require the synthesis of bespoke polymers, which limits their translation to existing process production methods and potentially compromises the traditional performance characteristics of polyolefins^18^. Oxo-degradable (sometimes also called oxo-biodegradable) plastics were also developed to promote polymer breakdown *via* oxidative mechanisms. However, these materials are often trialed under highly aggressive and unrealistic weathering cycles in the laboratory and have proven limited effectiveness in real-world environments. Consequently, the use of oxo-degradable and oxo-biodegradable plastics often results in incomplete degradation, limited mineralization, and the generation of microplastics^19,20^. Enzyme-based masterbatch technologies have also been proposed as an alternative strategy to enhance biodegradability; however, studies have reported only ~12% biodegradation over 6-month period, highlighting their limited performance in practical applications^21^.

Herein, we investigated the biodegradation potential in soil of PE and PP samples incorporating Polymateria’s Biotransformation Masterbatch technology (**PE-MB1,** **PP1-MB1**, and **PP2-MB2**). We report that the incorporation of these Masterbatches (MB) into PE and PP plastic materials leads to enhanced susceptibility to natural (bio)-degradation pathways, resulting in the formation of a low-molecular-weight, hydrophilic wax. The obtained wax was subjected to ecotoxicity and biodegradation tests in soil. Thus, it was found that the incorporation of Biotransformation Masterbatch technology in polyolefin-based materials led to desirable biodegradation characteristics through an abiotic and biotic biodegradation process, ultimately leading to full mineralization to CO_2_. Analysis of the soil after biodegradation also revealed that while biodegradable samples (cellulose and polyolefin waxes) did not lead to a significant taxonomic change in the microbial communities, they were enriched in metabolically active pathways compared to the virgin plastic controls. We envision that this technology can pave the way for broader adoption of biodegradable plastics in various applications as part of the solution to the challenge of plastic leakage in the environment.

## Results

### Accelerated laboratory weathering of masterbatch-incorporated polyolefin samples

In order to assess the degree of abiotic chemical transformation of the MB-incorporated polyolefin matrices (**PE-MB1,** **PP1-MB1** and **PP2-MB2**) over time, samples were aged in UV-accelerated conditions and the changes in carbonyl index (CI) and molecular weight (MW) were determined and assessed according to the BSI PAS 9017:2020 and equivalent standards^22^. The method for UV-accelerated weathering of the MB-incorporated polyethylene film sample (**PE-MB1**) involved a continuous, alternating cycle of 1 h of UV (0.80 ± 0.02 W/m^2^) exposure followed by 23 h in the dark at 60 °C for 14 days. This weathering cycle has been reported to be equivalent to 3 months in a tropical environment like South Florida^23^. For the UV-accelerated aging of MB-incorporated polypropylene rigid samples (**PP1-MB1** and **PP2-MB2**), samples were subjected to a continuous, staggered cycle of 8 h at 60 °C UV exposure (0.35 ± 0.02 W/m^2^) followed by 16 h in the dark at 60 °C for 28 days. Blank PP samples (without Biotransformation technology) were exposed to the same aging conditions for comparison.

**Fig. 1:**
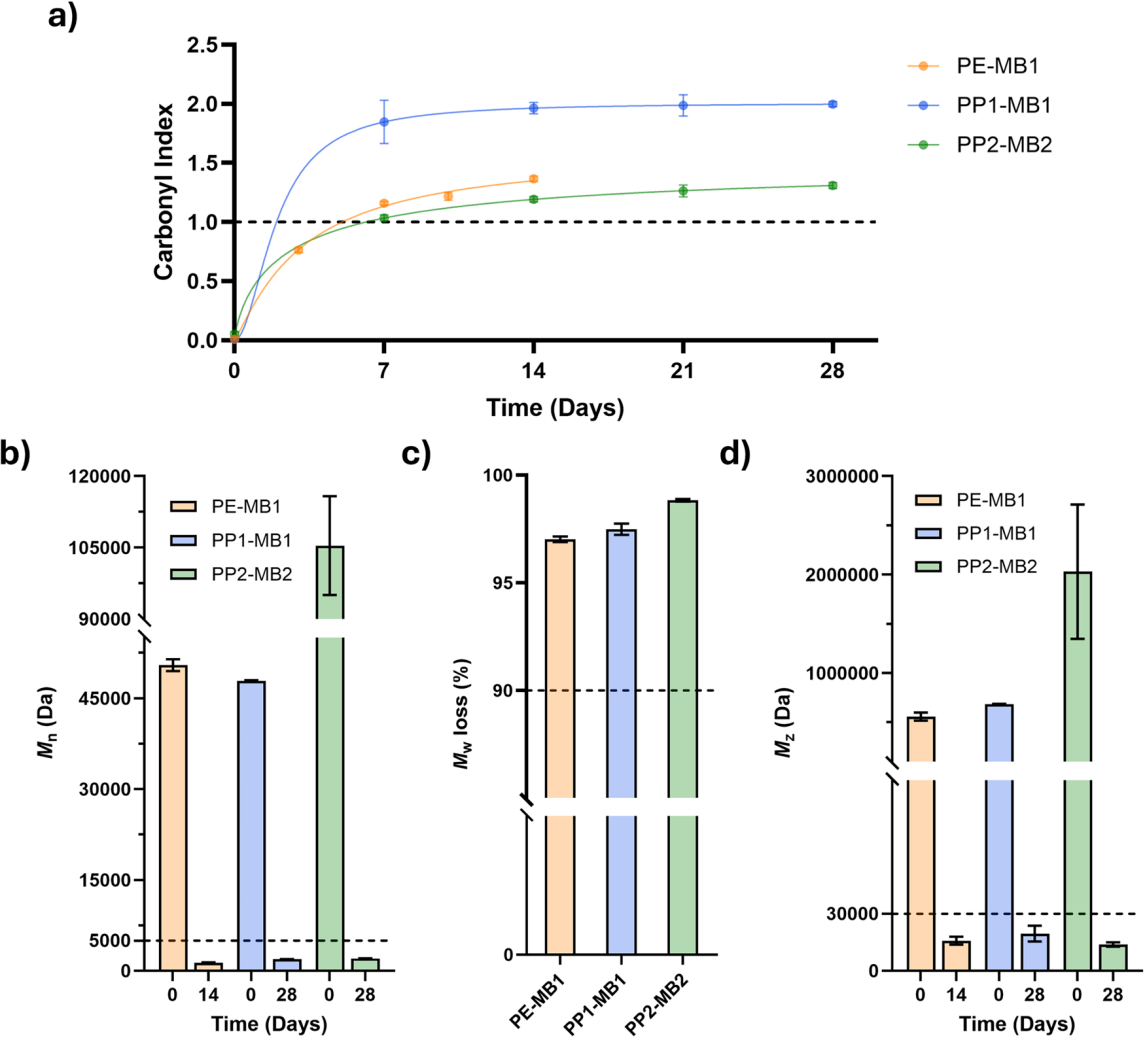
Accelerated laboratory weathering of MB-incorporated PE and PP samples. **a** The carbonyl index as a function of UV-aging time (14 days for **PE-MB1** or 28 days for **PP1-MB1** and **PP2-MB2**) obtained from ATR-FTIR analysis. Each point is an average with the standard error reported as bars (*n* = 3). Trend lines were fitted using a 4-parameter non-linear regression function. **b** Change in number average molecular weight (*M*_n_) after accelerated weathering, **c** % loss of the average molecular weight (*M*_w_) after accelerated weathering. **d** Change in z-average molecular weight (*M*_z_) after accelerated weathering. Dashed lines show the MW criteria for biodegradable polyolefins as defined in the BSI PAS 9017:2020 and equivalent standards.

Over this period, the degree of abiotic chemical transformation was assessed through CI and MW measurements to demonstrate the formation of carbonyl groups and chain scission of the polymer chains in the samples over time (Fig. 1). CI was measured using ATR-FTIR at various sampling intervals up to 28 days and calculated using the specified area under band (SAUB) methodology^24^. All samples reached a CI value above 1 within the timeframe, showing drastic chemical transformation in the polyolefin samples (Fig. 1a). Trend lines were fitted using a non-linear regression function, and the time to reach a CI value of 1 was determined through interpolation. The time it took **PE-MB1** to reach a CI value of 1 was calculated to be 5.03 days, whilst the time it took **PP1-MB1** and **PP2-MB2** to reach a CI value of 1 was 1.96 and 6.56 days, respectively. This is likely driven by the molecular structure of PP. Unlike PE, the PP polymer backbone contains tertiary carbon atoms, which have significantly lower carbon-hydrogen bond dissociation energies. This structural vulnerability results in a faster free-radical degradation cycle, leading to a rapid accumulation of carbonyl-containing and other degradation products (e.g., peroxide species). Because these products also absorb UV radiation, they create a feedback loop that further accelerates the degradation of PP relative to PE^25,26^.To further assess the degree of chemical transformation, the change in the MW of samples was assessed as an indication of polymer chain scission and associated property changes. Literature reports that polyethylene loses its plastic properties when *M*_w_ falls below 10,000–15,000 Da^27^ while ISO definitions highlight that plastic materials exhibit *M*_w_ above 10,000 Da^28^. In line with this, the BSI PAS 9017:2020 and equivalent standards define substantial chain scission in the polymer by a *M*_n_ value below 5000 Da, a loss in *M*_w_ of over 90%, and an *M*_z_ value of below 30,000 Da. Meeting these criteria signifies that the samples have transitioned from a plastic to a wax. All the MB-incorporated samples subjected to UV-aging study (**PE-MB1** over 14 days and **PP1-MB1** and **PP2-MB2** over 28 days) showcased a *M*_n_ value of below 5000 Da (Fig. 1b). **PE-MB1** showed a final *M*_n_ value of 1300 Da, whilst **PP1-MB1** and **PP2-MB2** exhibited a final *M*_n_ value of 1904 Da and 2042 Da, respectively. Correspondingly, >95% loss in *M*_w_ over the course of the aging study indicates effective polymer chain scission (Fig. 1c). Ultimately, an *M*_z_ value of below 30,000 Da was observed for all samples, with **PE-MB1** showcasing a final *M*_z_ value of 15,912 Da, whilst **PP1-MB1** and **PP2-MB2** exhibited a final *M*_z_ value of 19,629 Da and 13,880 Da, respectively (Fig. 1d). The final *M*_w_ values obtained (Supplementary Table 2) demonstrate the loss of the material’s plastic properties, resulting in a low-molecular weight, highly-carbonyl functionalized wax^29^.

Control experiments were conducted with **PP1** and **PP2** (without Biotransformation technology) in the same UV-aging conditions over the course of 52 days. After 52 days, blank **PP1** and **PP2** samples were visibly intact and did not show any signs of degradation. Compared to the UV accelerated-aging of **PP1-MB1** and **PP2-MB2** samples, the CI value did not reach a value of 1. Additionally, there was no significant MW decrease, suggesting minimal chain scission or breakdown of the polymer backbone (Supplementary Fig. 1, and Supplementary Table 3). This stark difference between control and MB-incorporated samples demonstrates that the Biotransformation Masterbatch technology promoted thermal and photolytic degradation. Introduction of carbonyl moieties and a rapid decrease in molecular weight suggest a free-radical chain mechanism generating hydroperoxide intermediates that break down quickly into carbonyl-containing species. The comparatively slow kinetic profile observed in the control samples indicates minimal rates of CI evolution, consistent with steady-state photooxidation conditions. The chemical transformations imposed are likely to have implications on polymeric microstructure, disrupting short- and long-range order of hierarchical organization in polyethylene and polypropylene samples. Oligomeric molecular weights increased short-chain branching and high carbonyl functionality stand to introduce structural defects throughout the microstructure. Whilst structural changes are not investigated explicitly herein, positive biodegradation results reported suggest the microstructural disruption that occurred is significant enough for microbial/bacterial consumption. These findings confirm the inherent stability of conventional polypropylene under accelerated UV-aging and underscore the critical role of the Biotransformation Masterbatch in promoting chemical transformation into wax.

### *Daphnia magna* reproduction test

In order to investigate the long-term effects of the wax obtained from weathered **PE-MB1,** **PP1-MB1**, and **PP2-MB2** samples on aquatic environments, *Daphnia magna* was used as a freshwater model invertebrate with the objective of assessing the chronic effects on the survival, growth, physiology, behavior, and reproduction according to OECD 211 guidelines^29^*. D. magna* is an important crustacean in freshwater ecosystems and commonly used for ecotoxicological assays because of its high sensitivity to external stressors, rapid reproduction, parthenogenetic reproductive nature, and ease of maintenance in the laboratory^30^.

**Fig. 2:**
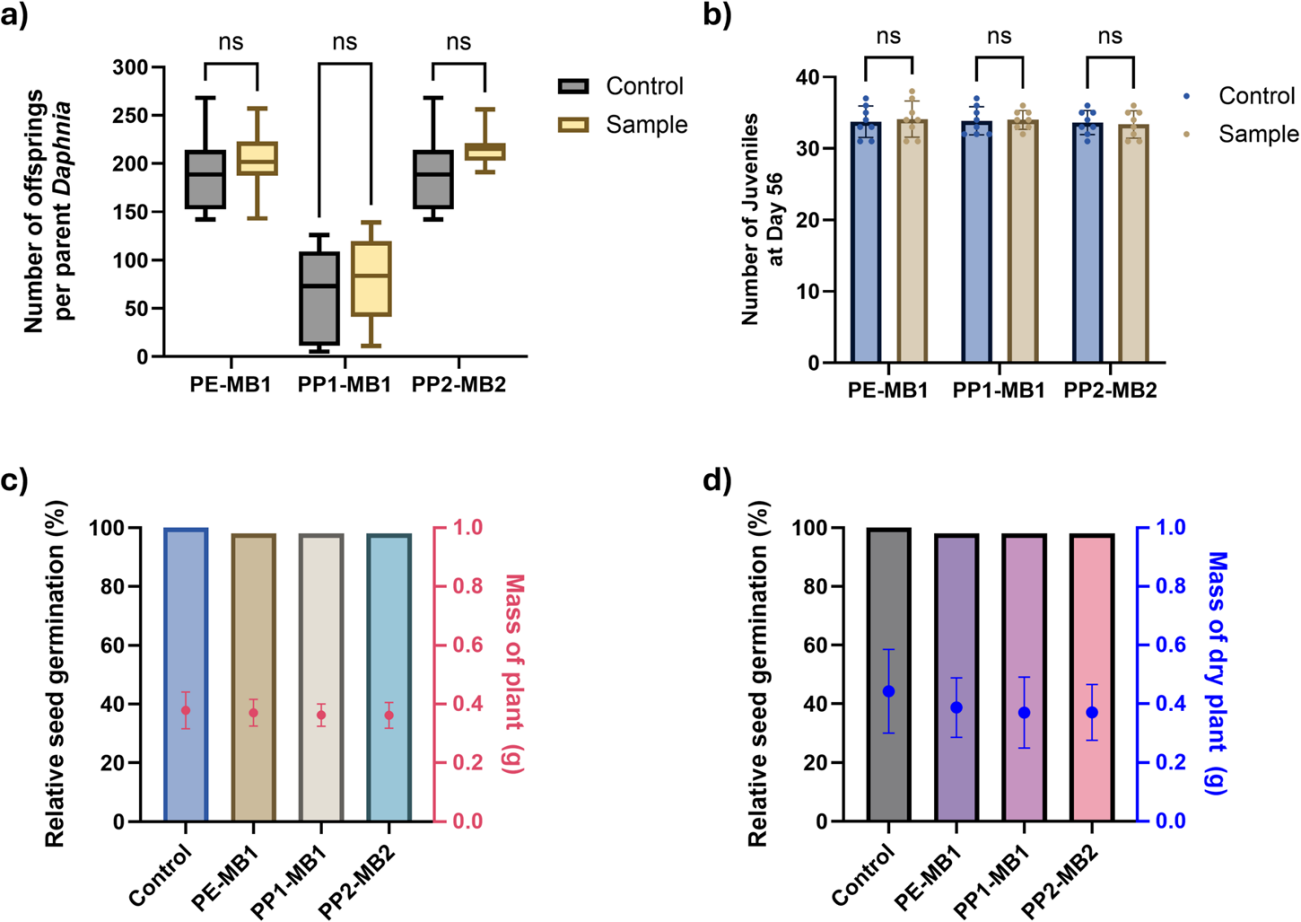
Ecotoxicity tests of PE-MB1, PP1-MB1 and PP2-MB2 samples after UV-accelerated weathering. **a** *Daphnia* *magna* reproduction test of samples at a concentration of 100 mg·L^−1^ (*n* = 10), **b** Earthworm reproduction test at a concentration of 1 g·kg^−1^ on dry weight basis added to soil (*n* = 8). Seedling emergence and growth test of the samples at a concentration of 1 g·kg^−1^ **c** on tomato (*Solanum lycopersicum*, *n* = 10) and **d** wheat (*Triticum aestivum*, *n* = 10). The samples consist of wax material obtained from MB-incorporated polyolefin samples following UV aging.

For this test, the Daphnia were subjected to 24 h leachates of 100 mg·L^−1^ of testing samples. Leachates were used to isolate and minimize the influence of physical particle size, thereby allowing the assessment to focus specifically on the ecotoxicological effects of chemicals leaching from the material^31^. In all cases, the filtered leachate of the wax and did not cause any mortality over the course of 21 days, suggesting no chronic toxicity of the plastic leachate to *D*. *magna*. The mean number of living offspring produced per parent animal after 21 days was above 60 in all instances (in line with the validity criteria of the test being ≥ 60 offsprings) (Fig. 2a). Compared to the controls (no treatment), wax leachates induced no statistically significant impact in reproduction (*p* > 0.05, *t* test). At the end of the test, the dissolved oxygen concentration was observed to be in the range of 7.16–8.97 mg·L^−1^, thereby confirming the validity of the test (validity criteria being >2 mg·L^−1^). Additionally, during the test period, daphnids remained mobile and consistently responded to gentle agitation by resuming swimming within 15 s after gentle agitation of the test beakers. Furthermore, test organism body lengths were measured over the course of the test and no abnormal effect on their growth was observed (Supplementary Fig. 2). Equivalent blank PP samples were also tested and showed no negative impact on mortality and reproduction rate (Supplementary Fig. 3). These results show that the Biotransformation technology leads to polyolefin waxes with no ecotoxicity on *D. magna* at the concentrations tested (100 mg.L^−1^).

### Earthworm reproduction test

An earthworm (*Eisenia fetida*) reproduction test was performed using the wax obtained from weathered **PE-MB1,** **PP1-MB1**, and **PP2-MB2** samples at a concentration of 1 g·kg^−1^ (on a dry weight basis) according to OECD 222 guidelines^32^. An average rate of production of juveniles was observed to be 33 over the course of 56 days in the control (validity criteria being ≥30 juveniles), and complete survival of all test organisms was observed in the control group throughout the experimental period, satisfying the validity criteria for the assay.

The average number of juveniles produced from earthworms treated with **PE-MB1** and **PP2-MB1** was measured to be 34 for both samples, while 33 juveniles were produced from the samples treated with **PP2-MB2**. A *t*-test of the results returned *p* > 0.05 in all cases, showing that the samples were not significantly different from each other. Thus, it can be concluded that no statistically significant negative effect on the reproduction of earthworms was observed when adding 1 g·kg^−1^ (dry weight basis) UV-aged polyolefin samples. Moreover, there was no statistically significant weight influence on the weight of the earthworms during the test, indicating no statistically significant impact on earthworm reproduction with exposure to the wax samples (Fig. 2b). Equivalent blank PP samples were also tested and showed no negative impact on mortality and reproduction rate (Supplementary Fig. 3). These results show that the Biotransformation technology leads to polyolefin waxes with no ecotoxicity at the concentrations tested (1 g∙kg^−1^).

### Seedling emergence and seedling growth test

In order to discern the impact of the wax obtained from weathered **PE-MB1,** **PP1-MB1**, and **PP2-MB2** samples on seed germination and seedling growth of tomato (*Solanum lycopersicum*) and wheat (*Triticum aestivum*), germination experiments were carried out according to OECD 208 guidelines^33^. The relative seed germination of tomato and wheat was not statistically significantly changed when exposed to the polyolefin wax samples. Compared to the control, all samples tested showed that they did not statistically significantly differ in seed germination rate (*p* > 0.05) under high-dose treatment of 1 g·kg^−1^. In addition, the results obtained indicated that there was no statistically significant change in biomass (dry-basis) of the plant; thus, the PE and PP waxes had no observable effect on the seed viability, and they did not impede with the growth of the plants (Fig. 2c, d).

Ecotoxicity assays were also conducted on blank PP controls (Supplementary Fig. 3). It was demonstrated that the presence of MB did not induce any significant ecotoxic effect compared to the blank, indicating that MB-incorporated samples remained environmentally benign.

### Biodegradation testing in soil

To evaluate the biodegradation behavior of the resultant wax obtained from UV-aged **PE-MB1,** **PP1-MB1**, and **PP2-MB2** samples, the ISO 17556 (2019) standard was followed^34^. Biodegradation progress was monitored through mineralization of the test samples, specifically by measuring CO_2_ production. The degree of biodegradation was calculated as the percentage of CO_2_ produced relative to the theoretical maximum CO_2_ that could be generated, and the results were plotted against time. According to the ISO 17556 method, the test is considered valid if the standard deviation of CO_2_ production in the control vessels is less than 20% of the mean, and the degree of biodegradation for the cellulose reference material must exceed 60% during the plateau phase or by the end of the test. In all cases, both conditions were met throughout the testing period.

**Fig. 3:**
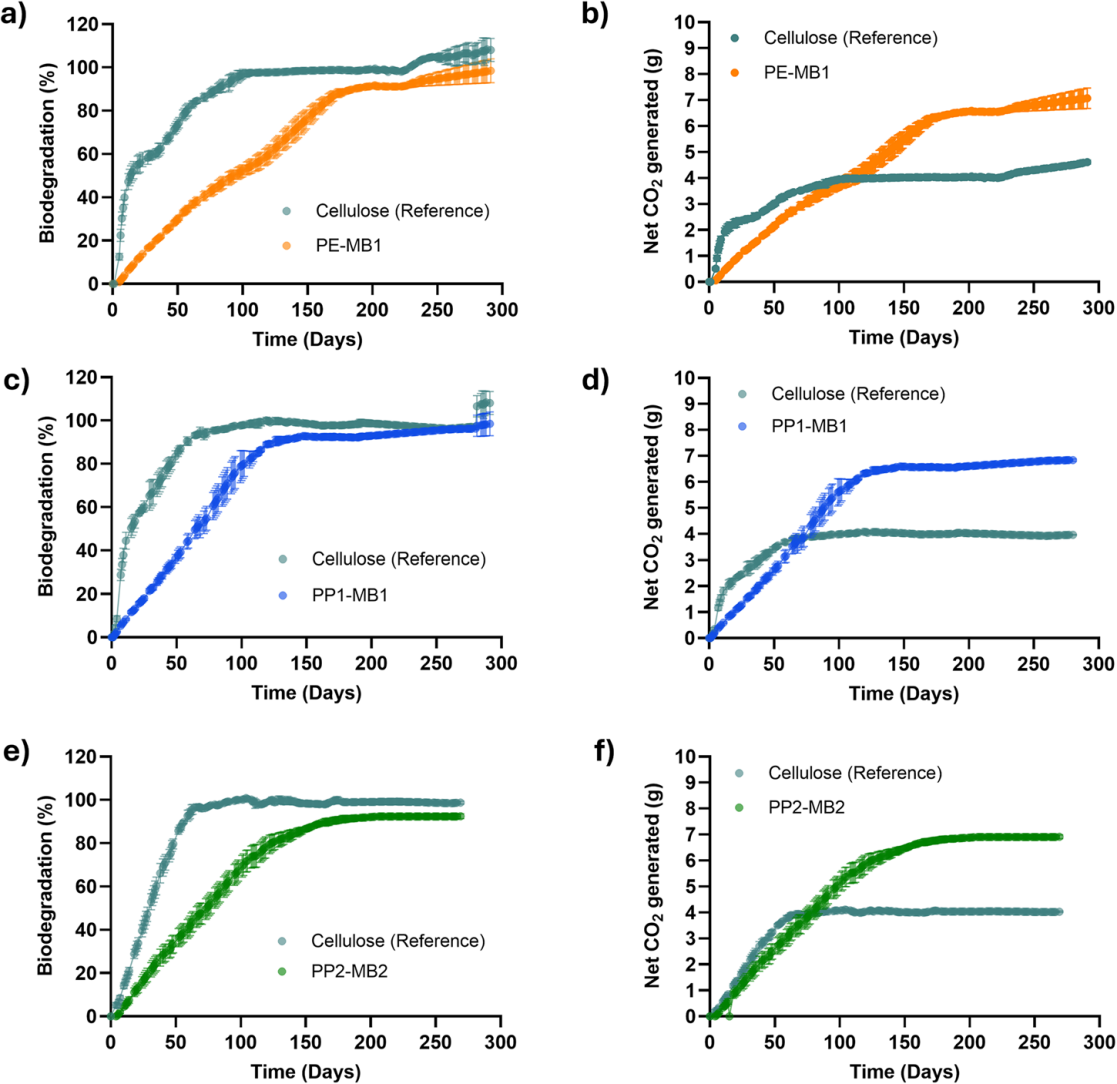
Biodegradation curves of **a** PE-MB1, **c** PP1-MB1 and **e** PP2-MB2 and reference substrate materials (cellulose). Net carbon dioxide (CO_2_) produced for samples **b** **PE-MB1**, **d** **PP1-MB1** and **f** **PP2-MB2** including reference substrate material (cellulose). The test was carried out at 28 ± 2 °C. Each curve is the mean of three replicates. The samples consist of wax material obtained from MB-incorporated polyolefin samples following UV weathering.

After 292 days, **PE-MB1** showed a cumulative biodegradation of 91.2% ± 3.8%, or 98.0% relative to the reference material, cellulose (Fig. 3a, b). For **PP1-MB1**, the biodegradation percentage reached 96.2% ± 0.7%, or 99.1% relative to cellulose, after 280 days (Fig. 3c, d). **PP2-MB2** achieved 92.4% ± 1.2% biodegradation, or 93.6% relative to cellulose, after 269 days (Fig. 3e, f). According to all globally recognized biodegradation standards, for a material to be considered completely biodegradable in soil, it must biodegrade by at least 90% absolute or relative to cellulose, within 720 days^22^. From the results observed above, it can be therefore concluded that the MB-incorporated polyolefin samples exhibited full biodegradability under aerobic conditions in mesophilic soil. We also assessed the biodegradation behavior of the PP virgin samples (without MB) and the results showed that UV-aged **PP1** and **PP2** samples were not biodegradable since they plateaued at 13.7% ± 1.6% (23.6% relative to cellulose) and 22.3% ± 1.28% (24% relative to cellulose) biodegradation after 180 days of incubation, respectively (Supplementary Fig. 4).

### Quantification of heavy metal content of tested materials

Due to the existence of residue or contaminant additives used in plastic production (e.g., plasticizers, stabilizers, pigments, etc.), the presence of heavy metals in plastic materials originating from these additives is under increasing regulatory scrutiny due to the health and environmental risks they can pose. European Directive 2002/72/EC, along with other international standards, has set strict limits on the permissible levels of heavy metals in consumer plastics^35–38^. For this reason, the concentrations of As, Cd, Cu, Cr, Mo, Ni, Pb, Se, and Zn in the both blank PP **(PP1** and **PP2)** samples and MB-incorporated samples (**PE-MB1,** **PP1-MB1** and **PP2-MB2**) were determined using inductively coupled plasma mass spectrometry (ICP-MS), while Hg was analyzed using a mercury analyzer, and fluorine content was measured via ion chromatography. In all tests, it was shown that concentrations of heavy metals and fluoride detected in the blank and MB-incorporated material were well below the standardized limits (Supplementary Tables 4 and 5).

### Microbial and metagenomic DNA analysis

Soil was collected at the end of biodegradation tests from all experimental vessels (cellulose, blank and polymers including waxes and conventional plastics) and subsequently analyzed by sequencing their metagenomic DNA to determine the microbial community abundance and functional structure. Shannon diversity indices ranged from 9.70 to 10.17 across all samples and did not differ significantly among treatment groups (Kruskal–Wallis, *p* = 0.17; Supplementary Table 6), indicating that treatments drove species turnover rather than richness loss.

**Fig. 4:**
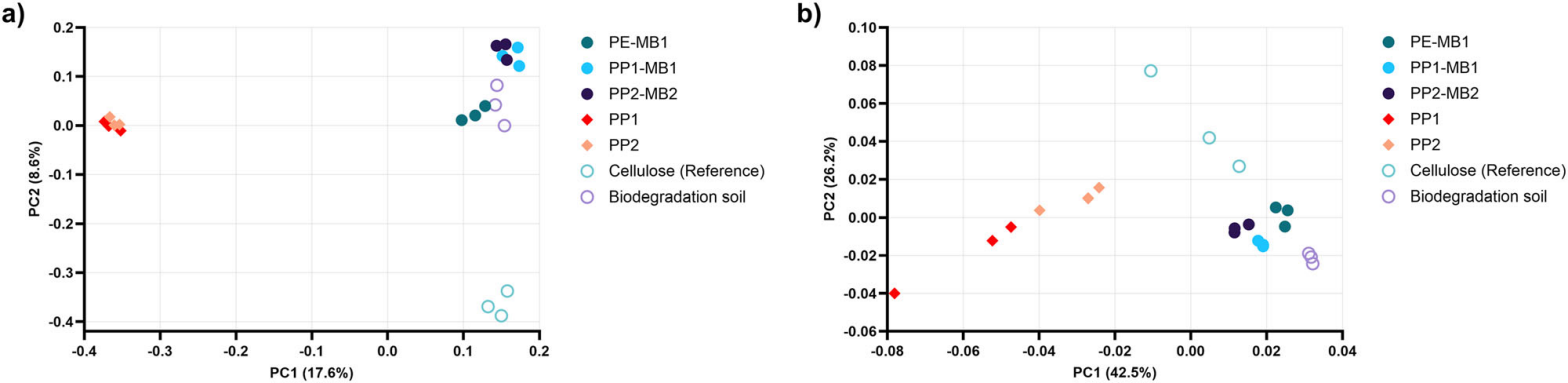
Principal Coordinate Analysis of Bray–Curtis dissimilarities between treatment groups based on **a** taxonomic composition (SingleM OTU abundances) and **b** functional composition (KEGG pathway abundances). PERMANOVA: **a** pseudo-*F* = 1.91, *R*² = 0.45, *p* < 0.001; **b** pseudo-*F* = 10.78, *R*² = 0.82, *p* < 0.001. PERMDISP: **a** *F* = 1.99, p = 0.004; **b** *F* = 1.64, *p* = 0.060. *n* = 3 biological replicates per treatment; 9999 permutations.

Principal Coordinate Analysis of Bray–Curtis dissimilarities revealed contrasting patterns at the taxonomic and functional levels (Fig. 4). Taxonomic composition showed moderate separation among treatments (Fig. 4a; 26.2% variance explained across two axes), with PERMANOVA attributing 45% of variation to treatment (pseudo-*F* = 1.91, *R*² = 0.45, *p* < 0.001), though significant dispersion heterogeneity was detected (PERMDISP *F* = 1.99, *p* = 0.004). Pairwise comparisons between individual treatments were non-significant after BH correction (all *p*_adj > 0.10). However, grouped contrasts revealed significant separation between MB and conventional plastic communities (*R*² = 0.23, *p*_adj = 0.003), while MB and blank soil communities were taxonomically indistinguishable (*R*² = 0.10, *p*_adj = 0.12).

On the other hand, functional profiles showed a stronger treatment structure (Fig. 4b; 68.7% variance explained). PERMANOVA attributed 82.2% of functional variation to treatment (pseudo-*F* = 10.78, *R*² = 0.82, *p* < 0.001) with no significant dispersion heterogeneity (PERMDISP *F* = 1.64, *p* = 0.060). This confirmed that the observed separation reflects genuine differences in functional composition between treatments rather than differences in within-group variability. All grouped pairwise comparisons were significant (all *p*_adj ≤ 0.015), including wax versus soil (*R*² = 0.53, *p*_adj = 0.010), which was absent at the taxonomic level. Hierarchical clustering of the 30 most abundant KEGG pathways confirmed this separation, with wax samples forming a distinct functional cluster from virgin plastics and cellulose controls (Supplementary Fig. 5). This taxonomic–functional decoupling, where communities exposed to low MW polymer waxes converge taxonomically toward unamended soil while diverging functionally, suggests that the bioavailability of the MB substrate drives metabolic restructuring without requiring wholesale compositional change.

**Fig. 5:**
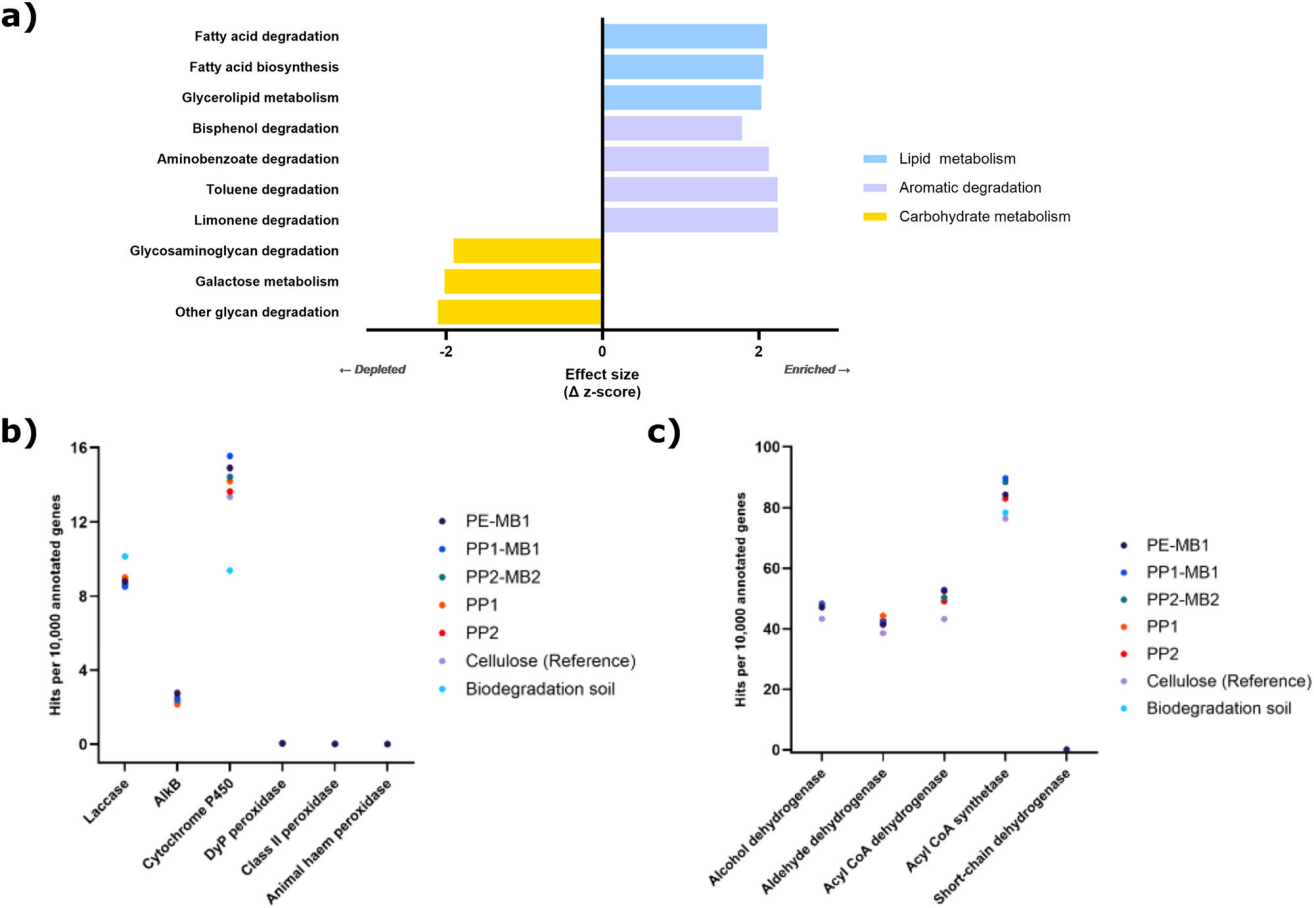
Differential functional profiles between wax (PE-MB1, PP1-MB1, PP2-MB2) and virgin plastic treatments (PP1, PP2). **a** Representative KEGG pathways with significant differential abundance between MB-incorporated (*n* = 9) and virgin plastic (*n* = 6) samples, grouped by functional category. Effect size represents the difference in mean z-score between groups; positive values indicate enrichment in MB treatments, negative values indicate depletion. Two-way ANOVA with Benjamini–Hochberg correction; all pathways shown are significant at FDR < 0.001. Full pathway results in Supplementary Fig. 6. **b** Abundance of oxidative enzyme families and **c** β-oxidation machinery across all treatment groups, quantified as Pfam domain hits per 10,000 annotated genes. Points represent treatment-level means from co-assembled metagenomes (*n* = 1 per treatment). Gene-level deduplication applied to resolve multi-domain inflation in laccases and PKS/NRPS mega enzymes.

To identify the specific metabolic pathways underlying the functional divergence observed in Fig. 4b, two-way ANOVA was performed on KEGG pathway abundances between wax and virgin plastic samples, identifying 245 pathways with significant differential abundance (FDR < 0.05; 143 enriched, 102 depleted; Supplementary Fig. 6). Representative pathways from the most coherent functional categories are shown in Fig. 5a. Lipid metabolism pathways (fatty acid degradation, fatty acid biosynthesis, and glycerolipid metabolism) were consistently enriched in MB samples, directly consistent with microbial catabolism of the low molecular weight, carbonyl functionalised hydrocarbon chains that constitute the waxy substrate. Pathways involved in aromatic compound degradation (aminobenzoate, toluene, limonene, and bisphenol degradation) were also enriched despite the absence of aromatic structures in the polymer waxes themselves. This suggests similarities in the oxidation pathways involved in the metabolism of hydrophobic recalcitrant molecules. Conversely, carbohydrate metabolism pathways (galactose metabolism, glycan degradation, glycosaminoglycan degradation) were depleted in MB samples relative to conventional plastic samples. This result could point towards a carbon utilisation niche displacement, as readily available MB-derived carbon reduces community reliance on naturally occurring soil polysaccharide substrates.

Given that oxidative enzymes are frequently cited in the literature as candidates for polyolefin backbone attack, Pfam domain abundances for key enzyme families were compared across all treatments (Fig. 5b). Cytochrome P450s and laccases were the most abundant oxidative families across all samples, but neither showed enrichment in MB or conventional plastic treatments relative to soil blanks (laccases were in fact most abundant in unamended soil, Supplementary Fig. 7). DyP-type peroxidases, Class II peroxidases, and animal haem peroxidases were essentially absent from all treatments except blank soil, where DyP peroxidases were detected at low abundance. β-oxidation machinery (alcohol dehydrogenase, aldehyde dehydrogenase, acyl-CoA dehydrogenase, acyl-CoA synthetase) was abundant and conserved across all treatments with no MB-specific enrichment, consistent with these enzymes representing core housekeeping functions rather than a substrate-induced response. The absence of enrichment in known oxidative enzyme families, combined with the strong lipid metabolism signal observed at the pathway level, indicates that microbial communities could be metabolising the bioavailable wax substrate through conventional fatty acid catabolism rather than through enzymatic attack on recalcitrant polymer backbones.

## Discussion

This study demonstrates that polyolefin materials (specifically PE and PP) incorporating Polymateria’s Biotransformation Masterbatch technology exhibit complete biodegradation in soil compared to untreated controls. Testing conducted in accordance with the BSI PAS 9017 and equivalent standards confirmed that (bio)-degradation follows the expected two-stage pathway, initial conversion of a polyolefin plastic into a low molecular weight, carbonyl functionalized polyolefin wax, followed by full microbial assimilation and conversion to carbon dioxide. Importantly, this work provides the first published evidence of complete biodegradation of polyolefins into CO₂, water, and biomass under relevant environmental conditions. Ecotoxicity assessments revealed no adverse effects in either soil or aquatic environments, underscoring the safety of the (bio)-degradation process. Metagenomics studies also demonstrated that the full biodegradation of these materials has no significant effects on the community composition at a taxonomic level compared to the untreated soil, although does show enrichment in specific metabolic pathways expected to be involved in the assimilation of aliphatic molecules, and diverges significantly from the changes resulting from treatment with a highly biodegradable and chemically less complex substrate as cellulose. Collectively, these findings validate the underlying mechanism of biotransformation and highlight the potential of this technology to address the environmental persistence of conventional plastics. In regions with limited waste management infrastructure, such innovation could play a pivotal role in mitigating plastic pollution, improving ecosystem health, and supporting a more sustainable approach to managing polyolefin waste globally.

## Methods

### Materials

The polyethylene film (**PE-MB1**) was manufactured using blown film extrusion, resulting in a multi-layer polyethylene film (80 µm thick) composed of 70% (w/w) LLDPE, 28% (w/w) LDPE, and 2% (w/w) Polymateria’s Biotransformation technology. The polypropylene rigid sample (**PP1-MB1**), with a thickness of 0.3–0.5 mm, was manufactured using injection molding. It consisted of 98% (w/w) polypropylene and 2% (w/w) Biotransformation technology (from the same batch as the PE film, **MB1**). The PP2-MB2 sample was fabricated via sheet thermoforming using a 300 µm thick sheet. It consisted of 98% (w/w) polypropylene and 2% (w/w) Biotransformation technology from a different masterbatch (**MB2**). Virgin polypropylene samples (**PP1** and **PP2**, without Biotransformation technology), identical in size, shape, and thickness, were used as control materials in accelerated laboratory weathering, ecotoxicity testing, and soil biodegradation experiments.

### Accelerated laboratory weathering

The accelerated weathering of the PE film was carried out by SIRIM QAS International Sdn Bhd in accordance with the specifications stated in standards, including BSI PAS 9017:2020, PNS 2166:2022; MSZ 29017:2022 or SIRIM ECO 098:2023^22^, using a QUV-A fluorescence tester from Q-Lab. An irradiance setpoint of 0.80 ± 0.02 W/m^2^ at 340 nm and black panel temperature of 60 ± 2 °C was set for 1 h light and 23 h dark cycle and repeated over 14 days. The accelerated weathering of **PP1-MB1,** **PP2-MB2** and blank PPs (conventional plastics) samples was carried out by SIRIM QAS International Sdn Bhd using a Q-SUN Xe‑3 tester according to ASTM standard D2565/ISO4892-2^39^. An irradiance setpoint of 0.35 ± 0.02 W/m^2^ at 340 nm with black panel temperature of 70 ± 2 °C for 8 h light cycle and black panel temperature of 60 ± 2 °C for 16 h dark cycle repeated over 28 days. Samples were allowed to condition at room temperature for 2 days before the FTIR and GPC analyzes were carried out.

### *D. magna* reproduction test

The experiments were designed following OECD 211 guidelines (*D. magna* Reproduction Test, 2012)^29^. The stock culture of *D. magna* was obtained from Freshwater Fisheries Research Centre (FFRC), Batu Berendam, Melaka, and the original culture was sourced from the Rijksuniversiteit Gent, Laboratorium voor Ecotoxicity, and maintained under controlled conditions at the Energy & Environment Centre (EEC), SIRIM Berhad. The organisms were kept in a permanent in-house breeding setup at 20 ± 1 °C with a 16 h light and 8 h dark cycle. Adult *Daphnia* were cultured in beakers containing 200 mL of holding water (modified M7 water prepared as described in OECD 211^29^), with ten healthy daphnids per beaker, following the conditions specified in the OECD 211 guidelines^29^. The cultured daphnids were fed daily with a 2.0 mL mixture of yeast and fish pellets. Neonate daphnids, aged less than 24 h, were selected for the study. For the reproduction test, 100 mg·L^−1^ stock solutions of the polyolefin samples in deionized water were prepared and shaken at 30 rpm at 23 °C for 24 h. After this, the solution was filtered through a glass filter with a pore size of 250 µm to remove any remaining particles. Healthy neonate daphnids were then exposed to 100 mL of the filtered sample solution for 21 days. The test was conducted in 250 mL glass beakers. Each parent animal was placed individually in a beaker containing 100 mL of the sample solution. The semi-static method was used, with ten animals held individually in each test container and ten animals held in the control group. The animals were fed daily, and the solution was not aerated. The test medium was renewed three times per week.

### Earthworm reproduction test

This study was conducted in accordance with OECD 222 guidelines (Earthworm Reproduction Test, 2016)^32^. *E. fetida* was selected as the model organism and subsequently cultured and tested at ToxIndia. UV-weathered **PE-MB1,** **PP1-MB1** and **PP2-MB2** samples were mixed with finely ground industrial quartz sand at a ratio of 1000 mg of sample per 10 g of sand. Two-liter glass beakers, covered with perforated plastic film, were used to contain 600 g of artificial soil composed of 10% (w/w) coco peat, 20% (w/w) kaolin clay, and 70% (w/w) fine industrial quartz sand. A total of 220 mL of distilled water was added to the artificial soil, resulting in a moisture content of 42–44%. The tests were performed with eight replicates for both the test concentration and control groups. Ten earthworms, each weighing between 385 and 450 mg, were added to each vessel, and the containers were incubated at a temperature of 19.1–21.2 °C under a light/dark cycle of 16:8 h (536–545 lx). The water content was checked gravimetrically once a week, and any evaporated water was replaced. Every 7 days, 5 g of uncontaminated food was added to the soil surface in each container. After 28 days, the adult earthworms were removed and weighed, and no additional food was provided for the remaining 28 days of the test. After 56 days, the number of juveniles in each test container was counted.

### Seedling emergence and seedling growth test

This study was conducted in accordance with OECD 208 guidelines (Terrestrial Plant Test: Seedling Emergence and Seedling Growth Test, 2006)^33^. Tomato and wheat seeds were obtained from Sin Seng Huat Seeds Sdn. Bhd. in Malaysia and maintained under good horticultural practices in a controlled environmental chamber and stored at room temperature. Polyolefin samples at 1 g·kg^−1^ were introduced in tomato and wheat plants. Ten replicates for each plant type, containing five seeds each, were prepared. Seeds were planted in each pot (150 mL), which would provide adequate growth conditions and avoid overcrowding for the duration of the test. The test duration for the tomato was 37 days, which included 4 days after 50% emergence of the control, whereas the test duration for the wheat was 40 days, which included 6 days after 50% emergence of the control. Relative seed germination was calculated as described by Luo et al.^40^.

1 $${\rm{Relative\; seed\; germination}}=\frac{{\rm{seeds\; germinated\; with\; test\; sample}}}{{\rm{seeds\; germinated\; with\; control}}}{\rm{x}}100$$

### Biodegradation test in soil

The ultimate aerobic biodegradability of the materials in soil was conducted according to the ISO 17556 (2019) standard^34^. For this study, the method used was based on gravimetric measurement of carbon dioxide (CO_2_) generation. The soil used was standardized soil (70% (w/w) quartz sand, 10% (w/w) kaolinite clay, 16% (w/w) natural soil, and 4% (w/w) mature compost). The natural soil was collected from the topsoil located within the SIRIM campus and the mature compost was obtained from a well-aerated compost from a municipal aerobic composting plant. The age of the compost was approximately 3 months old upon collection. The characteristics of the inoculum used at the beginning of the test are shown in Supplementary Table 1. The testing material was introduced in powder form (< 250 µm). 2.5 g (dry solids) of sample were mixed with 200 g (dry solids) in a 400 mL capacity reactor and maintained under optimum oxygen conditions, temperature 28 ± 2 °C and humidity (optimum water content between 40% and 60% of the total water holding capacity) until a plateau phase was observed. CO_2_-free air was supplied into the reactors to ensure aerobic conditions throughout the test. CO_2_ evolution in the exhaust air from the test vessels was monitored by tracking the change in an absorption column made of soda lime and soda talc trap. The percentage of biodegradation was calculated based on the net amount of carbon dioxide (CO₂) evolved from the test system. This was obtained by subtracting the CO₂ generated from the blank soil (m_B_) - which accounts for the background microbial activity - from the CO₂ generated in the presence of the test material (m_T_). The resulting value represents the CO₂ solely attributable to the biodegradation of the test substance. This net CO₂ evolution was then expressed as a percentage of the theoretical maximum amount of CO₂ (ThCO₂) that could be produced from complete mineralization of the test material. The calculation was performed using the following equation:

2 $$\% \,{Biodegradation}=\,\frac{\Sigma {m}_{T}-\Sigma {m}_{B}\,}{{ThC}{O}_{2}}\,x\,100$$

### DNA extraction, library preparation and DNA sequencing

Genomic DNA was extracted from samples using the DNeasy PowerSoil Pro Kit (Qiagen, USA) with mechanical lysis performed using a TissueLyzer II (Qiagen, USA) following the manufacturers protocol. DNA concentrations were quantified using a Qubit fluorometer (Thermo Fisher Scientific, USA). Sequencing libraries were prepared and sequenced by Azenta Life Sciences on an Illumina NovaSeq platform using 150 bp paired-end sequencing at an approximate depth of 10 Gbp per sample.

### Assembly, gene prediction and functional annotation

Raw sequencing reads were quality-filtered and adapter-trimmed using FastQC (v.0.12.1)^41^ and fastp^42^ (v1.0.1). For each treatment group (**PP1,** **PP2,** **PP1-MB1,** **PP2-MB2,** **PE-MB1**, soil and cellulose), quality-controlled reads from biological replicates (*n* = 3) were co-assembled using MEGAHIT (v1.2.9)^43^ with the meta-large preset and a minimum contig length of 500 bp, a strategy chosen to maximize assembly contiguity from low-biomass environmental samples. Open reading frames were predicted using Prodigal (v2.6.3)^44^ in metagenomic mode, and genes ≥100 bp were functionally annotated against the eggNOG database using eggNOG-mapper (v2.1.12)^45^, with KEGG Orthology and pathway assignments extracted from the mapper output and aggregated to pathway level. To identify candidate plastic-degrading enzymes, predicted protein sequences were additionally searched against PlasticDB^46^ and PAZy^47^ using DIAMOND (v2.1.13.167)^48^ with a minimum amino acid identity of 30% and query coverage of 50%. In addition, fourteen Pfam families with established or candidate roles in extracellular polymer oxidation and downstream β-oxidation were selected for further investigation. These were extracted from eggNOG-mapper output and include multi-copper oxidases (PF00394, PF07732, PF07731), DyP-type peroxidases (PF04261), AlkB alkane monooxygenases (PF00487), cytochrome P450s (PF00067), and β-oxidation enzyme families: aldehyde dehydrogenase (PF00171), alcohol dehydrogenase (PF00107), ADH_N domain (PF08240), Acyl-Coa dehydrogenase (PF00441), and AMP-binding Acyl-Coa synthetase (PF00501), short-chain dehydrogenase (PF00106).

### Read mapping, quantification and normalization

Quality-filtered reads from each biological replicate were mapped to their respective co-assemblies using Bowtie2 (v2.5.4)^49^ with default parameters. Per-gene read counts were quantified using featureCounts from the Subread package (v2.1.1) with the meta-feature level^50^. For KEGG pathway abundance analysis, raw read counts were normalised to counts per million (CPM) by dividing each gene’s count by the total mapped reads for that sample and multiplying by 10^6^, correcting for sequencing depth variation across samples and replicates. CPM-normalised counts were then summed across all genes assigned to each KEGG pathway to produce per-sample pathway abundance profiles. For targeted Pfam gene family analysis, gene abundances were normalised per 10,000 annotated genes, because assembly size and sequencing depth are decoupled in this dataset. Annotation rates were consistent across plastic-amended treatments (61–63%), with unamended soil showing marginally higher annotation (70%), likely reflecting the smaller but higher-quality gene catalogue recovered from the soil assembly due to the diversity-coverage trade-off in de Bruijn graph assembly. This difference is unlikely to introduce systematic bias into between-treatment comparisons.

### Taxonomic profiling and diversity analysis

Taxonomic profiling was performed independently of the assembly pipeline using SingleM (v0.18.1)^51^, which identifies taxa by extracting and clustering 14 conserved single-copy ribosomal protein marker genes directly from quality-filtered reads. Alpha diversity metrics (observed OTUs, Shannon index, Simpson index and Chao1) were calculated directly from SingleM OTU count tables without rarefaction. Sequencing depth was consistent across samples (mean ± SD = 98,818 ± 11,854 marker gene reads; CV = 0.12). Beta diversity was assessed using Bray–Curtis dissimilarity calculated from OTU relative abundance profiles, which are inherently depth-independent.

### Carbonyl index analysis using attenuated total reflectance Fourier transform infrared (ATR-FTIR)

ATR-FTIR spectroscopy was carried out on either a Nicolet iS10 or a Nicolet iS5 equipped iD7 Diamond ATR between 4000 cm^−1^ and 600 cm^−1^ at a resolution of 4 cm^−1^. The total carbonyl index was calculated using the SAUB method^24^ where CI = AUC_(1850 cm_−1_-1650 cm_−1_)_/AUC_(1500 cm_−1_-1420 cm_−1_)_. This method is recommended as it accounts for the full range of carbonyl species generated during weathering pathways.

### Molecular weight analysis using high temperature gel permeation chromatography (HT-GPC)

Molecular weights of the samples were carried out using an Agilent 1260 Infinity II High Temperature Gel Permeation Chromatography system equipped with Refractive Index detector, 3 × Olexis PL-Gel columns and calibrated using polystyrene (PS) standards. Samples were dissolved in 1,2,4-Trichlorobenzene with 0.001% (w/v) butylated hydroxytoluene at 160 °C for 4 h and measured at a flow rate of 1 mL·min^-1^. Molecular weight was calculated according to ASTM D6474-20^52^.

### Heavy metal analysis

Heavy metal analysis (As, Cd, Cu, Cr, Mo, Ni, Pb, Se, and Zn) was performed on the samples (**PE1,** **PP1,** **PP2,** **PE-MB1,** **PP1-MB1**, and **PP2-MB2**) using Inductively Coupled Plasma Mass Spectrometry (ICP-MS; PerkinElmer NexION 1000) following acid digestion in accordance with BS EN 1122^53^. Mercury (Hg) was analyzed using a Teledyne NIC Mercury Analyzer following the SW-846 Method 7473^54^. Fluorine analysis was conducted via Ion Chromatography according to APHA Method 4110 B^55^, after combustion and absorption of fluorine in deionized water using the Metrohm 930 Compact IC Flex system.

## Supplementary information

Supplementary information

## Data Availability

Raw sequencing reads and co-assembly metagenomes have been deposited in the European Nucleotide Archive (ENA) at EMBL-EBI under accession number PRJEB111165 (https://www.ebi.ac.uk/ena/browser/view/PRJEB111165). The datasets generated and/or analyzed during the current study are not publicly available due to institutional policies but are available from the corresponding author on reasonable request.
