# Supplementary material for: Masterbatch-enabled acceleration of polyolefin biodegradation under open air terrestrial environmental conditions
Source: Npj Mater Degrad. 2026 Aug 11;10(1):90. doi: 10.1038/s41529-026-00817-5 (PMC13461336; doi:10.1038/s41529-026-00817-5)
Supplement: Supplementary file 1 [file 41529_2026_817_MOESM1_ESM.pdf]

Masterbatch-Enabled Acceleration of Polyolefin Biodegradation under Open-Air Terrestrial Environmental Conditions

Ceren Kütahya[1†], Catalina Cruañas Pániker[2†], Faith Ly[1], Arjun Jerath[1], Emma Little[1], Rocio Gali[1], Taylor Young[1], Muhammad Zuhayr Bin Dzul Haimi[3], Abdul Hafiz Bin Abd Malek[3], Karimah Binti Muhamad[3], Suffeiya Binti Supian[3], Taylor Young[1], Sally Lawrence[1], Thomas Bell[4], Tan Yong Nee[3], Jose I. Jiménez[2,5] and Florence Huynh[1*]*

[1]Polymateria Limited, i-Hub, Imperial College White City Campus, 84 Wood Lane, London, United Kingdom

[2]Department of Life Sciences, Imperial College London, London SW7 2AZ, UK

[3]Standard and Industrial Research Institute of Malaysia (SIRIM), 1, Persiaran Dato' Menteri, P.O. Box 7035, Section 2, 40700 Shah Alam, Selangor, Malaysia

[4]Georgina Mace Centre for the Living Planet, Department of Life Sciences, Imperial College, London, SL5 7PY, UK

[5]Imperial Centre for Engineering Biology, South Kensington Campus, Imperial College London, SW7 2AZ, UK

[†] These authors contributed equally to this work.

* Corresponding author email: fh@polymateria.com, j.jimenez@imperial.ac.uk

Supplementary Table 1: Inoculum condition

**Characteristics**	**Inoculum**
Carbon	42.65 %
Hydrogen	4.22 %
C/N ratio	10.20
Total solid	30.44 %
Total volatile solid	69.68 %
Moisture content	30.32 %
pH	7.31 %
Total water-holding capacity	53%

Supplementary Table 2: MW change after accelerated laboratory weathering of MB-incorporated PE and PP samples[a]

**Time (Days)**	**PE-MB1**			**PP1-MB1**			**PP2-MB2**		
	M_n (kDa)	M_w (kDa)	M_z (kDa)	M_n (kDa)	M_w (kDa)	M_z (kDa)	M_n (kDa)	M_w (kDa)	M_z (kDa)
**28**	-	-	-	1.9	7.1	19.6	2.6	6.1	13.9
**14**	1.3	6.97	15.9	-	-	-	-	-	-
**0**	50.5	203.8	558.1	47.8	283.5	685.6	105.4	521.9	2030.4

[a]Samples were run at 160 °C in TCB containing 0.001% (w/v) BHT. MW values shown are average of two runs.

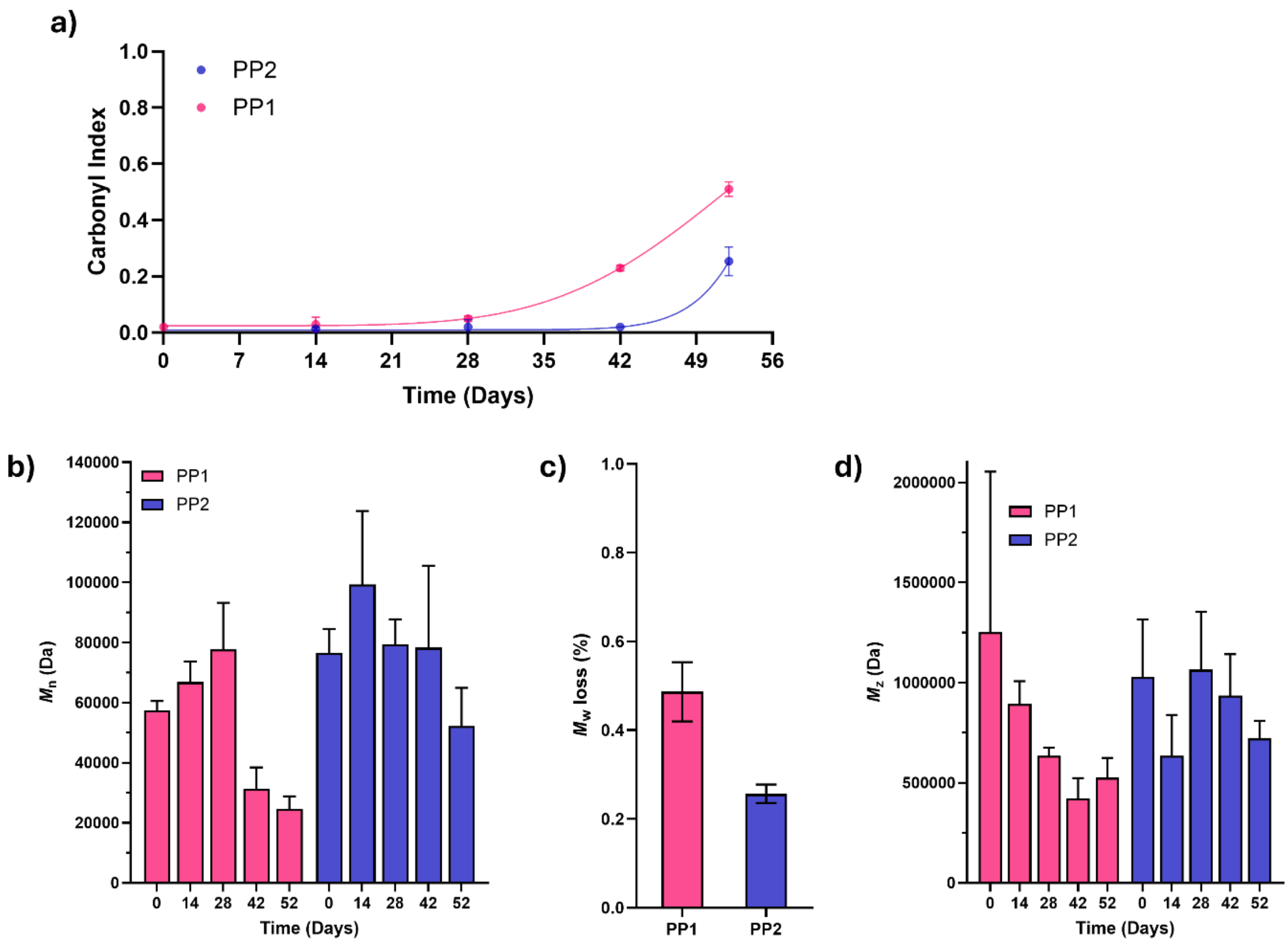

Supplementary Figure 1. Accelerated laboratory weathering of blank PP samples a) The carbonyl index as a function of UV-aging time over the course of 52 days obtained from ATR-FTIR analysis. Each point is an average with the standard error reported as bars (n = 3). Trend lines are fitted using a 4-parameter non-linear regression function, b) Change in number average molecular weight (M_n) after accelerated weathering, c) % loss of the average molecular weight (M_w) after accelerated weathering, d) Change in z-average molecular weight (M_z) after accelerated weathering.

Supplementary Table 3: CI and MW change after accelerated laboratory weathering of blank PP samples[a]

Time (Days)	PP1				PP2			
	CI[b]	M_n (kDa)	M_w (kDa)	M_z (kDa)	CI	M_n (kDa)	M_w (kDa)	M_z (kDa)
**52**	0.54	24.6	173.6	526.3	0.26	52.3	272.7	722.3
**42**	0.24	31.2	169.4	421.1	0.02	78.3	355.9	935.1
**28**	0.05	77.7	290.1	634.8	0.02	79.4	365.7	1064.5
**14**	0.03	66.9	335.7	893.3	0.01	99.3	297.3	636.3
**0**	0.02	57.4	338.9	1252.1	0.00	76.5	366.3	1028.7

[a]Samples were run at 160 °C in TCB containing 0.001% (w/v) BHT. MW values shown are average of three runs, [b]CI values shown are average of three runs and measured using ATR-FTIR at various sampling intervals up to 56 days and calculated using the SAUB methodology.

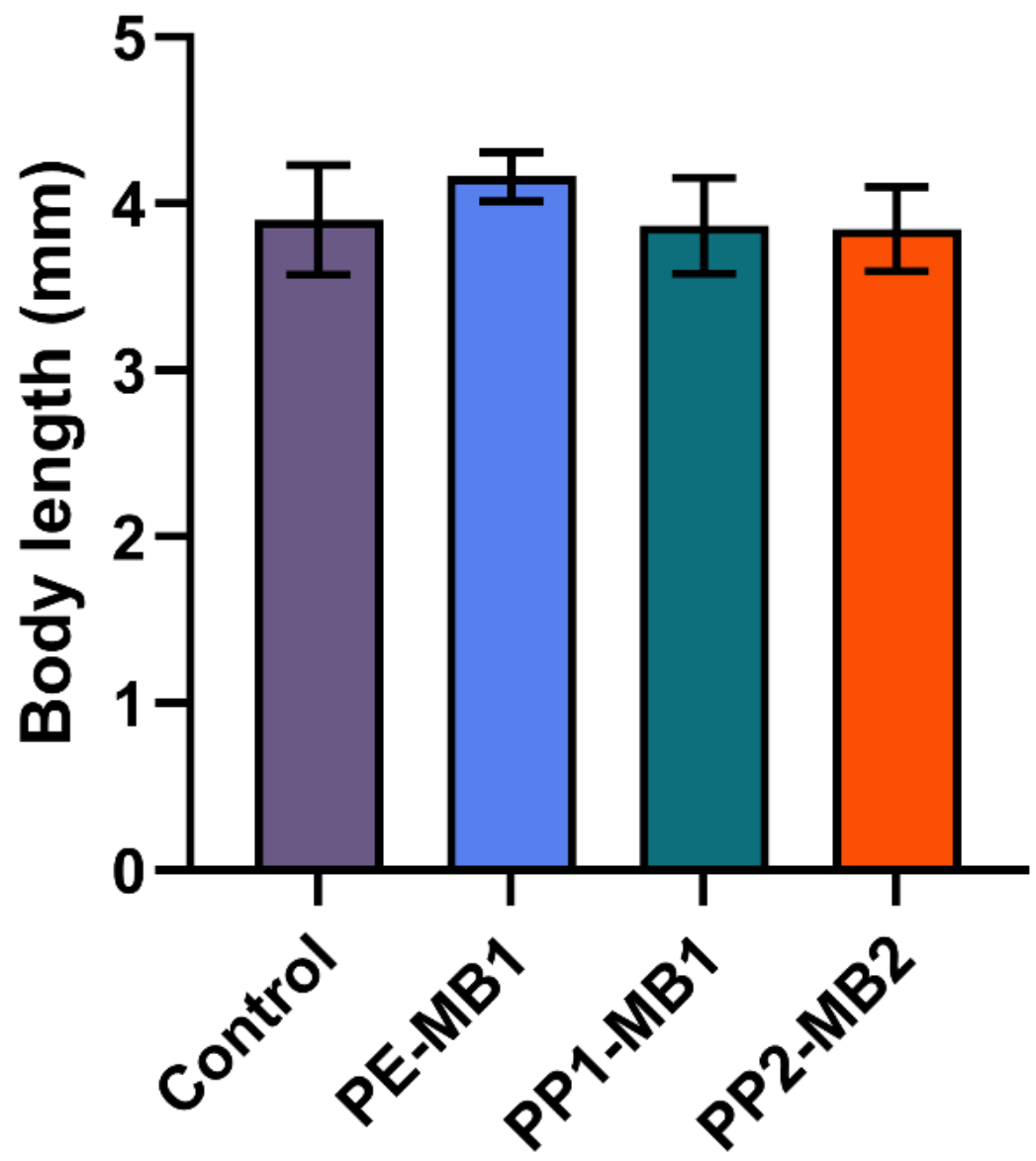

Supplementary Figure 2. Daphnia body lengths over the course of the test.

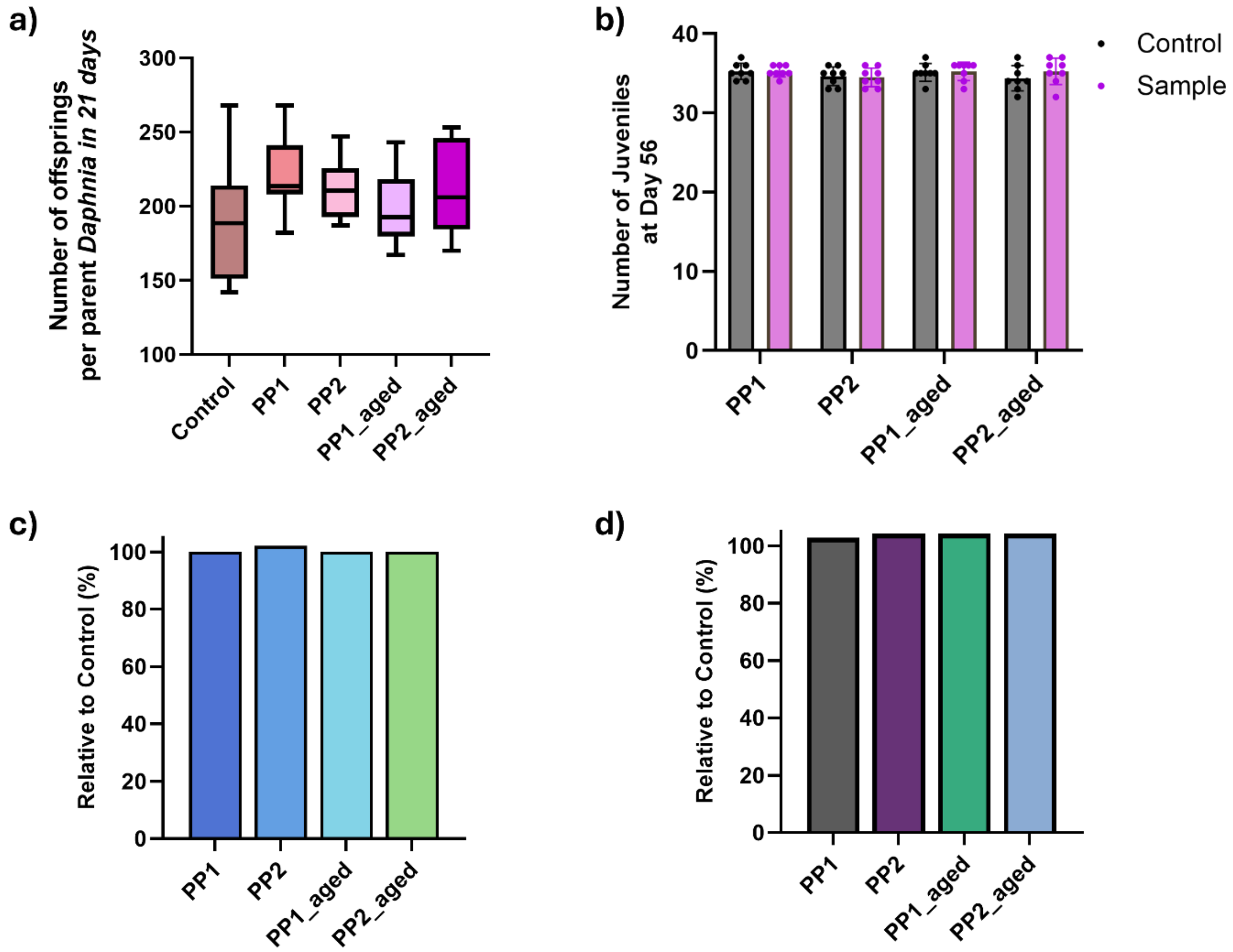

Supplementary Figure 3. Ecotoxicity tests of **PP1** and **PP2** samples after UV-accelerated degradation. a) Daphnia magna reproduction test of samples at a concentration of 100 $mg\cdot L^{-1}$ (n=10), b) Earthworm reproduction test at a concentration of 1 $g\cdot kg^{-1}$ on dry weight basis added to soil (n=8). Seedling emergence and growth test of the samples at a concentration of 1 $g\cdot kg^{-1}$ c) on tomato (*Solanum Lycopersi con*, n=10) and d) wheat (*Triticum aestivum*, n=10). The samples consist of blank (without MB) and aged materials following UV aging.

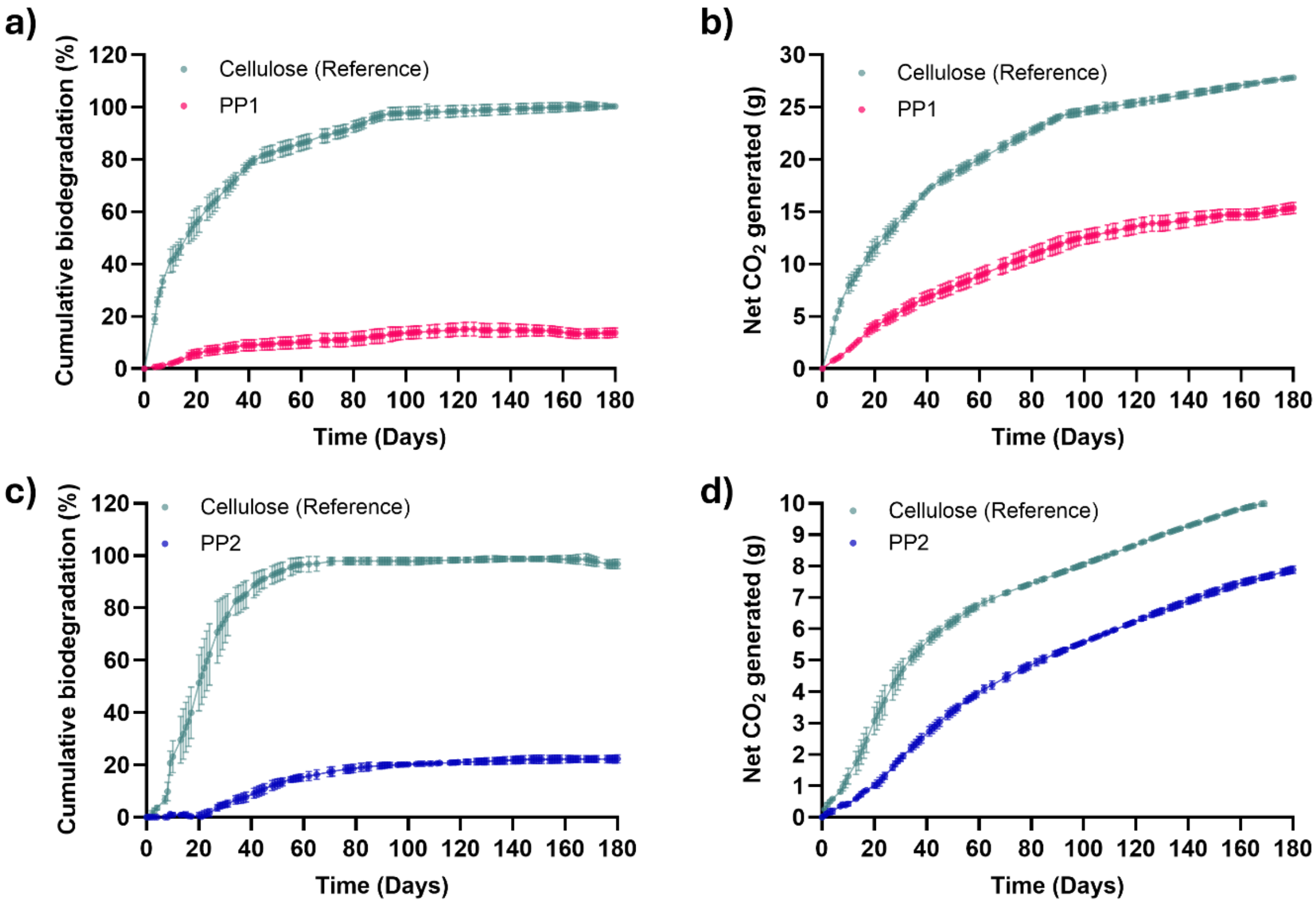

Supplementary Figure 4. Biodegradation curves of a) **PP1** and c) **PP2** and reference substrate materials (cellulose). Net carbon dioxide (CO_2) produced for samples b) **PP1** d) **PP2** including reference substrate material (cellulose). The test was carried out at 28 ± 2 °C. Each curve is the mean of three replicates.

Supplementary Table 4: Heavy metal content of tested samples[a]

Metal detected	PE-MB1 (ppm)	PP1-MB1 (ppm)	PP2-MB2 (ppm)	PP1 (ppm)	PP2 (ppm)
As	<0.01	<0.01	<0.01	<0.01	<0.009
Cd	<0.0001	<0.0001	<0.0001	<0.0001	<0.0002
Cr	1.9	0.4	0.7	0.3	0.5
Cu	0.3	<0.01	0.03	2.5	<0.02
Pb	<0.0004	<0.0004	<0.0004	<0.0004	<0.0005
Hg	0.01	0.02	0.01	0.01	0.01
Mo	0.7	0.4	0.6	0.5	0.5
Ni	1.0	<0.02	<0.02	<0.02	<0.02
Se	0.2	<0.005	<0.005	<0.005	<0.006
Zn	2.7	<0.01	<0.01	<0.01	<0.02
Fluorine	<0.001	<0.001	<0.001	<0.001	<0.001

[a]As, Cd, Cu, Cr, Mo, Ni, Pb, Se, and Zn in the tested materials were determined using inductively coupled plasma mass spectrometry (ICP-MS) after acid digestion according to BS EN 1122 method[1], Hg was analyzed using a mercury analyzer, and fluorine content was measured via ion chromatography.

Supplementary Table 5: Heavy metal content of wax samples after biodegradation test in soil[a]

Metal detected	PE-MB1[b] (ppm)			PP1-MB1[b] (ppm)			PP2-MB3[b] (ppm)		
	Blank	Cellulose	PE-MB1	Blank	Cellulose	PP1-MB1	Blank	Cellulose	PP2-MB2
As	6.6	4.3	4.8	3.1	5.5	5.7	2.6	4.2	3.2
Cd	<0.002	<0.002	<0.002	<0.002	<0.002	<0.002	<0.002	<0.002	<0.002
Cr	14.0	10.0	10.0	4.8	7.8	7.9	2.3	6.7	6.5
Cu	9.0	6.4	6.6	4.7	5.9	5.2	4.1	5.0	3.9
Pb	25.0	12.0	13.0	12.0	11.0	12.0	12.0	14.0	13.0
Hg	0.01	0.01	0.01	0.02	0.02	0.02	0.01	0.01	0.01
Mo	1.0	<0.0003	<0.0002	<0.0003	<0.0003	<0.0003	<0.0004	<0.0004	<0.0004
Ni	4.3	4.4	6.2	3.1	3.8	3.0	3.7	3.6	5.1
Se	<0.006	<0.006	<0.007	<0.006	<0.006	<0.006	<0.007	<0.007	<0.007
Zn	45.0	48.0	65.0	23.0	32.0	25.0	39.0	38.0	52.0
Fluorine	<0.001	<0.001	<0.001	<0.001	<0.001	<0.001	<0.001	<0.001	<0.001

[a]As, Cd, Cu, Cr, Mo, Ni, Pb, Se, and Zn in the tested materials were determined using inductively coupled plasma mass spectrometry (ICP-MS) after acid digestion according to BS EN 1122 method[1], Hg was analyzed using a mercury analyzer, and fluorine content was measured via ion chromatography. [b]The samples consist of wax material obtained from MB-incorporated polyolefin samples following UV aging.

Supplementary Table 6: Alpha diversity metrics across treatment groups[a]

Sample	Observed OTUs	SD	Shannon	SD	Simpson	SD	Chao1	SD
**PP1**	32844	4061	9.99	0.19	1	6.70 E-05	32843.8	4061.1
**PP2**	33612	2335	10.05	0.09	1	1.00 E-05	33612.2	2334.6
**PP1-MB1**	28996	2759	9.88	0.1	1	1.30 E-05	28996.4	2759.3
**PP2-MB2**	26175	3034	9.77	0.09	1	1.10 E-05	26174.7	3034.4
**PE-MB1**	27719	2277	9.83	0.04	1	8.30 E-06	27719.2	2277.1
**Soil**	30746	2729	9.9	0.1	1	9.30 E-06	30745.8	2729
**Cellulose**	28573	2294	9.9	0.06	1	7.40 E-06	28573	2294.1

[a] Values represent standard deviation across biological replicates (n = 3 per treatment). Metrics were derived from SingleM taxonomic profiles at a consistent sequencing depth (C = 0.12). No significant differences in alpha diversity were observed among treatments (Mann–Whitney U test, adjusted $p > 0.05$ for all pairwise comparisons).

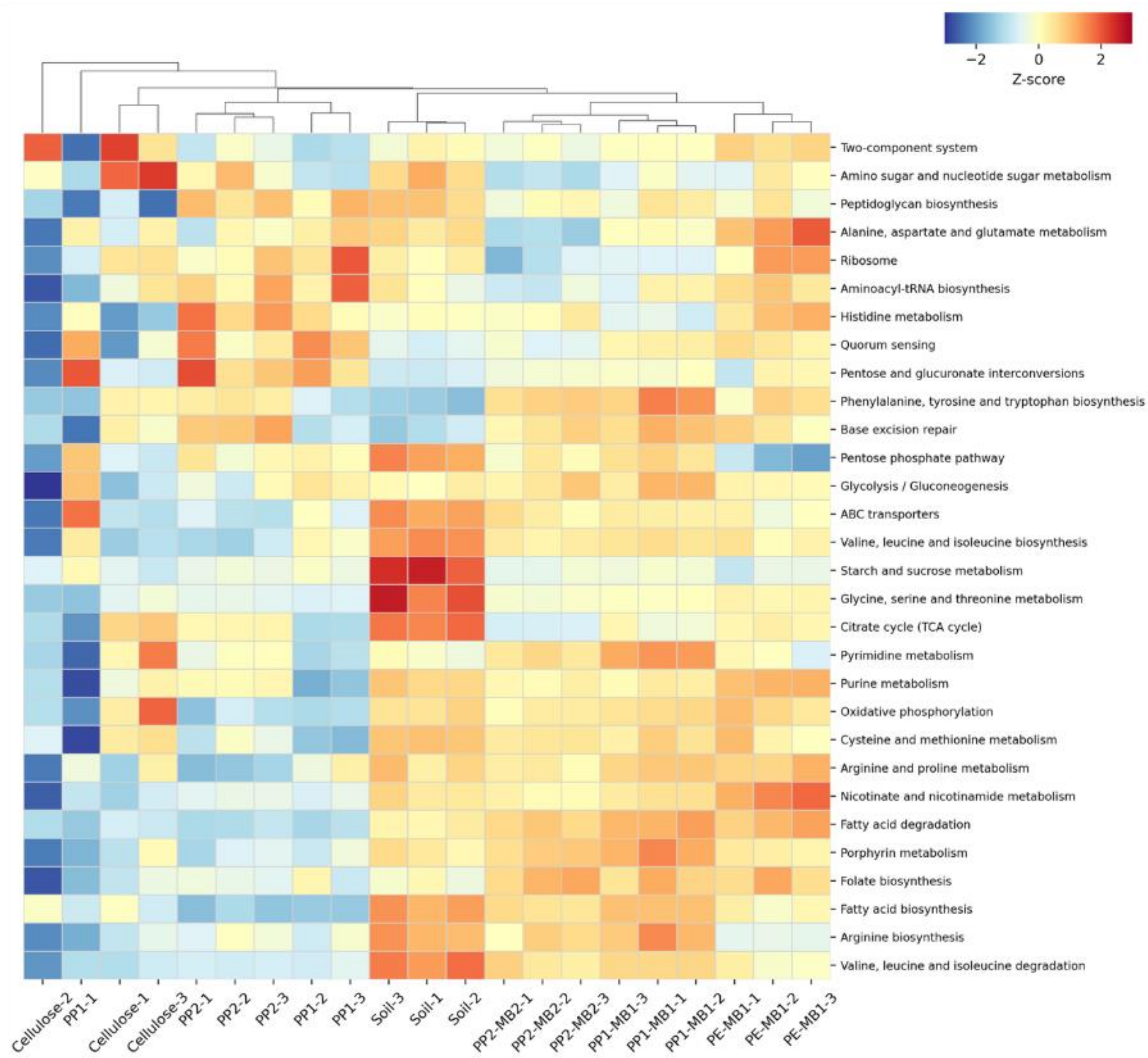

Supplementary Figure 5. Functional clustering of the top 30 most abundant KEGG pathways across all sample replicates. Z-score normalised KEGG pathway abundances show distinct clustering by polymer biodegradability.

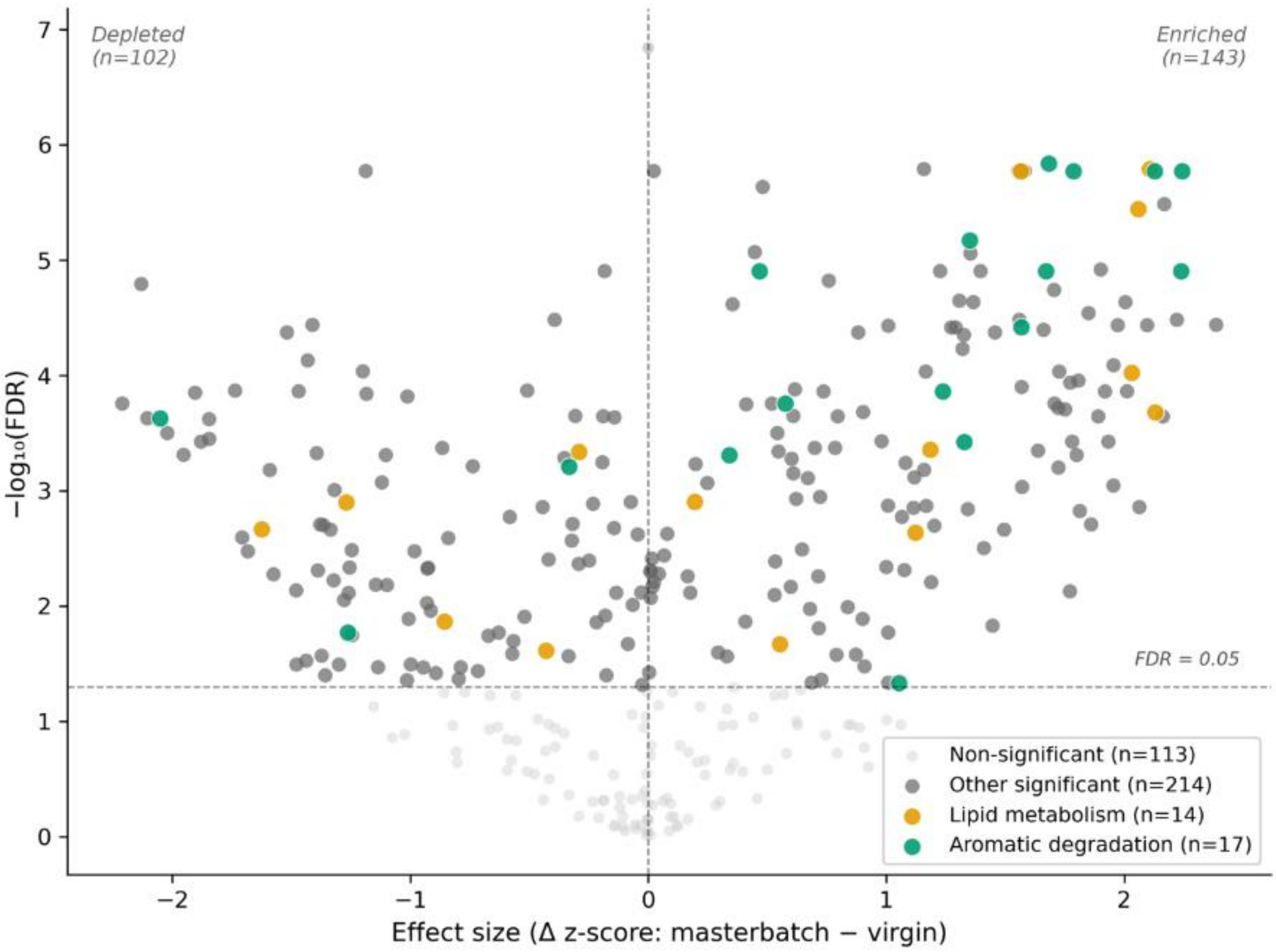

Supplementary Figure 6. Volcano plot of KEGG pathway differential abundance between MB samples (n = 9) and conventional plastic (n = 6) samples. Each point represents one pathway; x-axis shows effect size (Δ z-score: mean MB − mean conventional plastic), y-axis shows statistical significance ($-\log_{10}$ FDR). Horizontal dashed line indicates FDR = 0.05. Of 358 pathways tested, 245 were significant (FDR < 0.05), comprising 143 enriched and 102 depleted in MB relative to conventional plastic. Lipid metabolism (orange) and aromatic degradation (teal) pathways highlighted; representative pathways from these categories shown in Figure 5a. Two-way ANOVA with Benjamini-Hochberg correction.

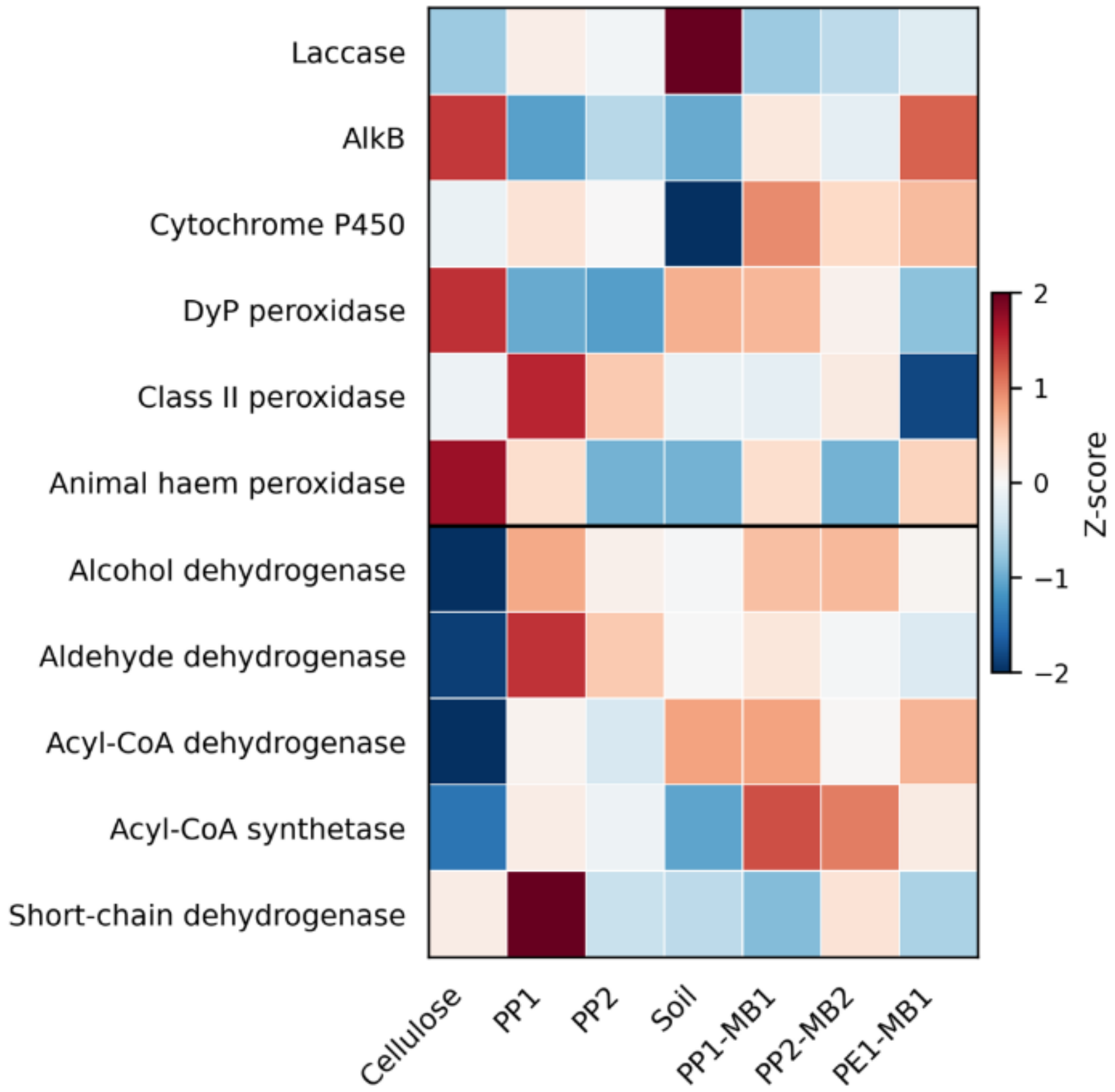

Supplementary Figure 7. Z-score heatmap of Pfam domain abundances for oxidative enzyme families (top) and β-oxidation machinery (bottom), normalised per 10,000 annotated genes. Columns ordered by treatment group: cellulose, virgin plastics (PP1, PP2), biodegradation soil, and MB waxes (PP1-MB1, PP2-MB2, PE-MB1).Gene-level deduplication applied to resolve multi-domain inflation in laccases (three Cu-oxidase domains per gene) and PKS/NRPS mega-enzymes.

References

1. Plastics. Determination of cadmium. Wet decomposition method. (2001).
